# Early MRI response monitoring of patients with advanced hepatocellular carcinoma under treatment with the multikinase inhibitor sorafenib

**DOI:** 10.1186/1471-2407-9-208

**Published:** 2009-06-28

**Authors:** Marius Horger, Ulrich M Lauer, Christina Schraml, Christoph P Berg, Ursula Koppenhöfer, Claus D Claussen, Michael Gregor, Michael Bitzer

**Affiliations:** 1 Department of Diagnostic Radiology, Eberhard-Karls-University, Hoppe-Seyler-Str. 3, D-72076 Tübingen, Germany; 2 Department of Gastroenterology & Hepatology, Medical University Hospital, Eberhard-Karls-University, Otfried-Müller-Str. 10, D-72076 Tübingen, Germany

## Abstract

**Background:**

New therapeutic principles in clinical oncology require the adjustment of response criteria to govern therapy decisions. For advanced hepatocellular carcinoma (HCC) a new era has recently begun by the approval of the multikinase inhibitor sorafenib. As a unique feature, HCC usually develops in a diseased liver and current imaging technologies employing classical response criteria have not been prospectively evaluated for this new treatment.

**Methods:**

MRI signal patterns were assessed in 21 advanced HCC patients receiving sorafenib. MRI was performed at baseline and in short-term intervals thereafter. Signal changes under therapy on T1WI, T2WI and post-gadolinium images including necrosis volume and its ratio to the entire tumor volume were compared to baseline imaging. To assess the association between the categorical variables, Fisher's exact tests were applied for a statistical analysis. Survey time ranged from 2–65 weeks, and a total of 39 target lesions were evaluated.

**Results:**

Signal abnormalities during sorafenib therapy were disclosed by T1WI and T2WI in 15/21 patients. The predominant tumor signal change was hyperintensity on both T1WI and T2WI. Interestingly, most patients developed MRI signal changes within 4 weeks of therapy; in contrast, two non-responders did not show any signal alteration at follow-up. Under therapy, 16/21 patients presented with new or progressive necrosis, whereas 7 patients achieved temporarily >75% tumor necrosis under sorafenib. Significantly associated MRI variables were increase in T1WI signal and tumor necrosis (p = 0.017) as well as increase of tumor necrosis with an elevated ratio of necrotic to vital tumor areas (p = 0.002). Remarkably, some (3/13) of the patients developing necrotic tumor areas showed a relevant (>20%) increase in tumor volume, which should be considered in the assessment of imaging studies.

**Conclusion:**

As sorafenib induces early intralesional necrosis with profound changes in T1WI/T2WI MRI signal intensities and measurable necrotic tumor areas in most HCC patients, early MRI-based evaluation could pave the way for its rationale and cost-effective application.

## Background

Hepatocellular carcinoma (HCC) is the fifth most common malignancy worldwide, with approximately 500,000 new cases per year [1,2]. Patients with unresectable tumor manifestations or metastatic disease exhibit median survival times of only a few months [3]. Sorafenib, which is a novel oral multikinase inhibitor [4], presents a promising new therapeutic option for HCC, as demonstrated in preclinical and recent clinical phase II and III studies by Abou-Alfa *et al. *[5] and Llovet *et al. *[6], respectively. Sorafenib hits tumor cells on multiple levels such as the Raf/MEK/ERK signalling pathway, as well as angiogenesis by targeting vascular endothelial growth factor receptors-1/-2/-3 (VEGFR-1/-2/-3) or platelet derived growth factor receptor beta (PDGFR-β) tyrosine kinases [7-9]. Based on the highly encouraging findings in a phase III trial [6], sorafenib has been approved recently for the treatment of unresectable advanced HCC. In this context, it is of utmost importance to define non-invasive tools, which are suitable for monitoring and governance of this novel therapy.

During HCC therapy, the special environment of primary liver tumor lesions presents a unique condition that has to be carefully taken into account when evaluating results of diagnostic imaging procedures such as CT or MRI. Moreover, it has been questioned whether "classical" or the recently revised Response Evaluation Criteria in Solid Tumors (RECIST) criteria alone [10,11] are reliable for the assessment of new molecular therapeutics [12-15].

The rationale of our MR-imaging observation study was to identify and define early MR-signal abnormalities in HCC patients receiving sorafenib monotherapy, which could be used as potential response criteria to monitor and govern the new treatment approach of multikinase inhibition in HCC.

## Methods

### Demographics

Between September 2005 and May 2008, 21 patients (20 men, 1 woman; age range, 42–77 years; mean, 64.2 years) with inoperable HCC, no prior systemic treatment, and Child-Pugh class A or B, received continuous oral sorafenib in 4-week cycles (Table 1). Three patients were investigated in part during the phase III SHARP trial [6]. The other patients were investigated according to the local standards for patients at our institution. The institutional review board approved this retrospective study and waived informed consent.

**Table 1:** Baseline characteristics with age, gender, histology including tumor differentiation and Child-Pugh score.

Patient	Age	Gender	**Histology**^a^	**Histological differentiation**^b^	Child-Pugh
**1**	58	male	HCC	+	A
**2**	59	male	HCC	+	A
**3**	65	male	HCC	-	B
**4**	72	male	HCC	++	A
**5**	65	male	HCC	++	A
**6**	65	male	HCC/CC	+/-	-^c^
**7**	67	male	HCC	+	B
**8**	63	female	HCC	++	A
**9**	71	male	HCC	++	A
**10**	65	male	HCC	++	A
**11**	43	male	HCC	++	A
**12**	77	male	HCC	+	A
**13**	54	male	HCC	+	A
**14**	65	male	HCC	++	A
**15**	66	male	HCC	+	A
**16**	65	male	HCC	-	A
**17**	61	male	HCC	+	A
**18**	76	male	HCC	-	A
**19**	59	male	HCC	-	B
**20**	65	male	HCC	n.d.^d^	A
**21**	69	male	HCC	+	A

aHCC/CC means a tumor with characteristics of both, hepatic and biliary differentiation.

bHistological differentiation: (++) good, (+) moderate and (-) poor tumor differentiation type.

cNo cirrhosis according to the histology of the liver.

dNo histology available; however, AFP value 1.591 μg/l and typical noninvasive HCC criteria

according to current imaging guidelines.

### Treatment Plan

Patients received sorafenib 800 mg daily (2 × 400 mg bid), but were allowed up to two dose reductions (400 mg per day or 400 mg every other day/200 mg per day) or a temporary interruption in case of drug-related toxicities. Treatment continued until disease progression (PD) or unacceptable toxicities. Dose delays or modifications were required for drug-related grade 3/4 toxicities; patients received lower doses when toxicity improved to grade 2 or better, but therapy was discontinued if recovery time was 3 weeks or longer. MR-parameters did not affect the clinical decision of a further sorafenib treatment.

### MR technique

MR-investigations were performed at baseline (pre-treatment) and follow-up (first MRI after a median of 3 weeks (range, 2–5 weeks); second MRI after a median of an additional 5 weeks (range, 3–9 weeks); subsequent MR-studies were performed every 8 weeks (range, 6–8 weeks). Overall, a total of 48 MR-investigations were performed and evaluated.

All patients received baseline MR-scans of the liver; in patients with metastases additional MR-scans of the chest or pelvis were performed. All scans were performed using the same 1.5 T whole-body unit (Magnetom Avanto/Espree, Siemens Medical Solutions, Erlangen, Germany). The body coil was used for radiofrequency transmission and the flexible matrix body coil in combination with the spine matrix was used for signal detection. For the Gd-enhanced MR-imaging, gadopentetate dimeglumine (Magnevist; Bayer Vital GmbH, Leverkusen, Germany) was administered as an intravenous bolus injection (0.1 mmol/kg) at 2 ml/s followed by a saline flush and image acquisition 2 min later.

The MR-protocol included axial T1-weighted images (T1WI), T2-weighted images (T2WI), Gadolinium (Gd)-enhanced dynamic sequences (VIBE) and additional post-contrast imaging. T1WI were acquired before and after contrast media administration with a standard spoiled gradient echo sequence (FLASH, 2D encoded). Sequence parameters: TR 205 ms, TE 4.1 ms, flip angle 70°, bandwidth 140 Hz/pixel. Chemical-shift selective fat suppression was used. Slice thickness 5 mm, FoV 360 × 270 mm^2^, matrix size 256 × 134, voxel size 2.0 × 1.4 × 5.0 mm^3^. A 3D fat-suppressed VIBE sequence was obtained before and 15, 30, 60 and 120 s after the start of the IV contrast administration. Due to the slightly different number of slices needed for whole liver coverage depending on the organ size, the length of each VIBE-sequence was 15–20 s. Thus, the second VIBE sequence was performed ca. 30–35 s after contrast administration whereas the other two VIBE sequences were performed 60 and 120 s after the contrast was IV given. T2WI images were acquired with a fast spin echo sequence (TR 1250 ms, TE 96 ms, flip angle 150°, bandwidth 300 Hz/pixel, echo spacing 7.4 ms, fast spin echo factor 13). Chemical-shift selective fat suppression was used. Slice thickness 5 mm, FoV 360 × 270 mm^2^, matrix size 320 × 180, voxel size 1.5 × 1.1 × 5.0 mm^3^. Additional coronal HASTE (TE/TR, 1100/118 ms) images of the liver were performed. A delayed contrast-enhanced axial fat-suppressed FLASH sequence was performed 2 min after the dynamic study (ca. 5 min after the start of the IV contrast administration).

### Response Assessment by Size and Volume Measurements

For the decision whether sorafenib therapy was continued or stopped due to progression under treatment, response was assessed for every MR-scan based on investigator-assessed one-dimensional tumor *size *measurements according to modified RECIST criteria [10]. In case of a confluence of several tumor lesions the joint diameter was considered for further measurements. Only one patient (#15) presented with ill-defined extra-hepatic osseous HCC-manifestations that could not reliably be measured at follow-up. To verify investigator observations in an unbiased manner, an independent assessment of the MR scans was performed retrospectively for all patients.

Additionally, entire *tumor volumes *and the *volume of *subsequent necrosis within the tumors were calculated by three-dimensional measurements (in cm^3^). Next, the ratio of the necrosis volume [NV] in relation to the entire tumor volume [TV] was calculated (percentage of [NT]/[TV]) and expressed as a score: <25%, <50%, <75%, or >75 (Table 2). Occurrence or progression of a central or diffuse decrease or absence of Gd-enhancement with subsequent signal hypointensity was considered typical for tumor necrosis.

**Table 2:** MRI signal and tumor characteristics of HCC target lesions at baseline and follow-up

Pat.	Dose **(mg)**^#^	MRI-FU	Lesion (no.)	Size (mm)	T1	T2 fs	Necr.	[NT]/[TV] (%)	Sum (mm)
**1**	-	baseline	liver	25	2	4	-	-	25
	800	3 w.	(1)	30	4	4	↑	<25	30
	800	8 w.		30	2	5	↑	<25	30
	800	16 w.		34	2	5	↑	<25	34

**2**	-	baseline	liver	160	2	3	+	<50	170
	400	2 w.	(2)	165	4	5	↑	<50	175
	800	6 w.		160	4	3	↑	<75	172
	400	14 w.		155	2	3	↑	>75	172

**3**	-	baseline	liver	35	2	4	-	-	35
	800	2 w.	(4)	35	2	4	-	-	35
	200	6 w.		35	2	4	-	-	35
	200	12 w.		39	4	3	↑	<25	39

**4**	-	baseline	liver	57	3	4	+	<25	57
	800	4 w.	(1)	57	4	5	↔	<25	57

**5**	-	baseline	liver	30	2	4	+	<25	30
	800	2 w.	(1)	30	2	4	↔	<25	30
	800	6 w.		31	2	5	↔	<25	31
	400	13 w.		30	2	5	↔	<25	30
	400	20 w.		40	2	5	↔	<25	40

**6**	-	baseline	liver	133	2	4	+	<25	133
	800	3 w.	(1)	135	3	5	↑	<25	135
	800	7 w.		130	2	3	↔	<25	130

**7**	-	baseline	liver	38	2	2	+	<25	70
	800	2 w.	(3)	45	5	4	↑	>75	80
	-	8 w.		32	3	3	↓	<50	66
	400	16 w.		32	3	3	↓	<25	71

**8**	-	baseline	adrenal	45	3	3	+	<25	144
	800	3 w.	(3)	45	5	4	↑	<25	144
	400	8 w.		57	5	4	↑	<25	156

**9**	-	baseline	liver	20	2	4	-	-	119
	800	5 w.	(3)	22	4	5	↑	>75	123
	800	14 w.		20	4	5	↑	>75	119
	800	20 w.		20	2	4	↑	>75	119

**10**	-	baseline	perit.	12	2	3	-	-	12
	800	2 w.	(2)	12	3	5	↑	+*	12
	800	7 w.		6	3	3	↔	+*	6
	400	15 w.		0	-	-	-	-	0
	400	23 w.		0	-	-	-	-	0

**11**	-	baseline	liver	10	2	4	-	-	19
	800	3 w.	(2)	14	2	4	-	-	26
	800	7 w.		17	2	4	-	-	31
	800	15 w.		18	2	4	-	-	35
	800	22 w.		21	2	4	-	-	38

**12**	-	baseline	adrenal	15	2	4	+	<25	50
	800	4 w.	(2)	12	2	4	↑	<50	42
	800	12 w.		8	2	5	↑	<50	36
	200	19 w.		8	2	5	↔	<50	38

**13**	-	baseline	liver	38	3	4	+	<25	38
	400	3 w.	(1)	38	3	3	↑	<25	38
	-	7 w.		55	4	3	↑	<25	55
	200	15 w.		39	3	3	↑	<50	39

**14**	-	baseline	liver	17	5	2	+	<25	70
	800	2 w.	(2)	17	5	2	↔	<25	70
	800	5 w.		17	5	2	↑	<50	70
	200	15 w.		17	5	2	↔	<50	70
	400	23 w.		17	5	2	↑	>75	70

**15**	-	baseline	bone	45	4	2	-	-	149
	800	5 w.	(3)	55	5	4	↑	>75	176
	800	9 w.		45	4	5	↔	>75	193
	800	16 w.		44	4	4	↔	>75	172
	800	21 w.		45	5	4	↔	>75	168
	800	24 w.		50	5	3	↔	>75	163
	800	32 w.		60	4	3	↔	>75	172

**16**	-	baseline	liver	52	4	2	+	<25	83
	400	3 w.	(2)	52	4	2	↔	<25	83
	-	9 w.		52	3	2	↔	<25	83

**17**	-	baseline	liver	51	1	4	+	<25	93
	800	4 w.	(2)	57	4	5	↑	>75	107

**18**	-	baseline	liver	120	1	4	+	<50	120
	800	7 w.	(1)	130	1	4	↓	<50	130

**19**	-	baseline	liver	151	1	4	+	<25	201
	400	6 w.	(2)	155	4	2	↑	<50	210
	800	18 w.		165	5	5	↑	>75	229

**20**	-	baseline	liver	15	3	3	-	-	15
	400	2 w.	(1)	15	5	4	↑	<50	15

**21**	-	baseline	liver	62	2	4	-	-	88
	400	8 w.	(2)	75	4	5	↑	<50	97
	200	15 w.		75	4	5	↔	<50	97
	200	22 w.		81	3	3	↔	<50	96
	200	29 w.		98	5	5	↓	<50	113

Abbreviations:

Pat.: patient number. #Daily dose at the time point of each MRI imaging study. MRI-FU: MRI at follow-up;

w.: week after therapy initiation. Lesion: location of representative analysed lesion and number of total lesions at this location. Size: size of the representative tumor lesion im mm. T1: T1-weighted image. T2fs: T2-weighted fat saturated image. Qualitative assessment of signal in relation to the signal of liver parenchyma (1 = markedly hypointense; 2 = slightly hypointense; 3 = isointense; 4 = slightly hyperintense; 5 = markedly hyperintense).

PV: portal vein; perit.: peritoneal. Gd: Gadolinium-enhancement at baseline and its evolution at follow-up

(↑ = increase; ↓ = decrease; ↔ = unchanged; (-) no necrosis detectable; (+) necrosis at baseline detectable). [NT]/[TV]% = percentage of necrosis volume to total tumor volume, expressed as a score with (--) no necrosis, <25%, <50%, <75%, or >75%. Sum (mm) = sum of the diameter of all detectable lesions at this location.

+* = the necrotic areas could not be reliably quantified.

### Response Assessment by Tumor Signal Changes

The investigation of the sorafenib treated patients was not simply based on tumor size measurements but included additional MRI parameters: the assessment of signal abnormalities occurring in the tumor on T1WI and T2WI as well as on post-gadolinium images. These parameters were employed to monitor the development of hemorrhagic necrosis, thus providing an insight into the morphologic and functional intratumoral changes under therapy. Lesions that underwent previous local therapy (RFA, TACE, etc.) were excluded from the analysis. Lesions with a diameter of <1 cm, that remained stable during the study and were not histologically proven to be malignant, were not evaluated because of the limited ability of MRI to reliably resolve small structures. For image interpretation, signal characteristics on T1WI and T2WI were compared to the signal of adjacent normal liver by visual assessment according to an established grading described by van den Bos *et al. *[16]: 1 = markedly hypointense; 2 = slightly hypointense; 3 = isointense; 4 = slightly hyperintense; 5 = markedly hyperintense. The gold standard for assessment of response to sorafenib monotherapy was the joint clinical and MRI follow-up, according to RECIST.

### Statistical Methods

Tumor lesions were classified for presence or absence of increased T1 signal, tumor necrosis and increase in necrotic volume to the entire tumor volume [NV/TV]. Fisher's exact tests were used to assess the association between categorical variables, whereas p < 0.05 was regarded a significant relationship between the classification factors.

## Results

Patients' baseline characteristics with respect to age, sex, and tumor histology including differentiation and Child-Pugh score are shown in Table 1. Information to sorafenib doses at follow-up is included in Table 2.

### Response Assessment by Measurement of Size and Volume

In 38% of our patients, tumor lesions experiencing morphological signs compatible with tumor necrosis under sorafenib showed at least a temporary increase in tumor size, up to 58% of the initial size of the lesion, suggesting that this might reflect an increased volume of liquid tumor parts. In 3 patients volume increase of HCC was found to be over the 20% threshold stipulated by RECIST, in 3 other patients volume increase was >10% of the initial lesion's volume (Table 2). In 3 patients (#1, #13, and #15) a substantial reduction of the whole tumor volume could be achieved under sorafenib (reduction of 36%, 45%, and 30%, respectively). Of note, two patients (#7, #21) undergoing long- and short-term sorafenib dose interruptions experienced repeated responses with temporal variations in tumor volume.

### Response Assessment by Measurement of Tumor Necrosis

On post-gadolinium fat-saturated T1WI, areas of tumor necrosis appeared *de novo *in 7/21 patients (Table 2) and 13/39 lesions (data not shown) or increased in 9/21 patients (Table 2) and 9/39 lesions (data not shown). In patients with progressive necrosis 2 lesions developed <25% necrosis, 4 lesions achieved <50% necrosis, and 7 lesions developed >75% tumor necrosis at follow-up. Altogether, progression of necrosis (↑) was diagnosed in 40/117 followed-up lesions (data not shown). Notably, reduction (↓) of the volume of tumor necrosis was registered only in 3 patients (#7, #18, and #21). The former two had progressive disease at follow-up with revitalization of tumor. The latter presented an undulant course of the disease due to intermittent dose discontinuations.

### Assessment of T1WI and T2WI signal changes as a surrogate marker for intratumoral hemorrhage

A focal or diffuse increase in tumor signal to baseline, confirmed independently by both readers, was detected on nonenhanced T1WI in 15/21 patients (Table 2) and 28/39 target lesions analysed (data not shown) at an early time point after a median of 5 weeks (range, 2–9 weeks) following onset of sorafenib therapy, and considered suspicious of hemorrhage and/or protein-rich necrosis. Interestingly, one patient with a complete response (#10) displayed early signal changes in T1WI, T2WI and an increase of necrotic areas within the tumor already 2 weeks after initiation of sorafenib therapy 400 mg bid. Increased signal on T1WI returned to normal with time, but the dynamics of this phenomenon could not be entirely analysed due to different time spans of the patients' individual MR-surveys. In five patients (#5, #11, #12, #14, #18), the T1WI tumor signal remained unchanged during follow-up. However, two of them developed an increase of the T2-signal (#5, #12) while in two patients a substantial progression of the necrotic tumor area could be detected (#12, #14) at time.

A synchronous signal increase on *T2WI SE-sequences *or rarely a temporary signal decrease was considered as further supporting the hypothesis of tumor hemorrhage. Almost synchronously with early signal intensity changes on T1WI, signal abnormalities on T2WI were found (median, 5.3 weeks; range, 2–12 weeks), consisting in most of the cases (15/21 patients) of an increase in signal intensity (Table 2) and in 26/39 target lesions analysed (data not shown), whereas a decrease in T2-signal lesions was found in only 2/21 patients. In two patients (#5, #12) with stable signal patterns on T1WI, signal changes could be assessed only on T2WI. Thus, early signal abnormalities suggestive of tumor hemorrhage occurring after onset of sorafenib therapy were encountered in 15/21 patients.

**Figure 1:**
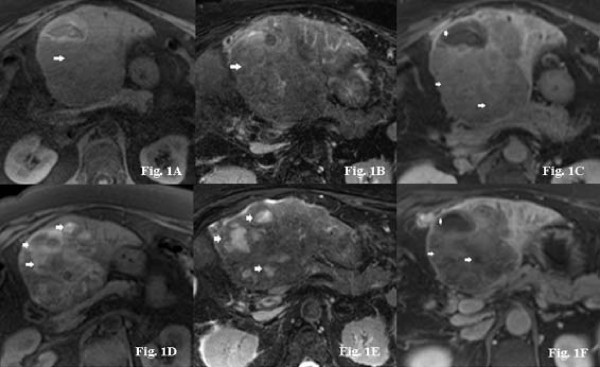
**A-F. 63-year-old female patient with multicentric HCC (patient #8)**. Axial nonenhanced T1WI performed at baseline showed a 10 cm large tumor (arrow) in the left liver lobe (Figure 1A). Note a tumor signal (arrow) slightly hypointense to normal liver parenchyma with a small ventral heterogeneous hyperintense area caused by earlier radiofrequency (RFA) ablation. On baseline T2WI, the tumor revealed diffuse mild hyperintensity and a small hypointense area corresponding to the ablation site (Figure 1B). Baseline fat-suppressed post-gadolinium (Gd) imaging demonstrated diffuse tumor enhancement (arrows) with focal necrosis due to the earlier RFA procedure (arrowhead) (Figure 1C). Three weeks after onset of sorafenib, T1WI imaging detected multiple focal hyperintense lesions (arrows) in part with sedimentation levels that have occurred during therapy (Figure 1D). On T2WI at the same time, corresponding hyperintense lesions were seen (arrows) (Figure 1E). Fat-suppressed post-Gd imaging revealed extensive necrosis (arrows) and reduction in tumor perfusion (Figure 1F).

**Figure 2:**
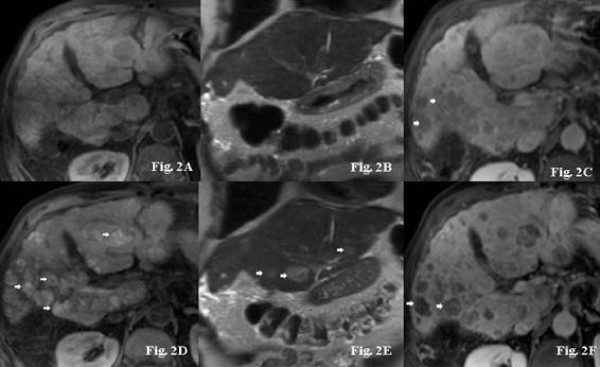
**A-F. 71-year-old male patient with multifocal HCC (patient #9)**. Axial nonenhanced fat-suppressed T1WI imaging of the liver performed at baseline showed multiple mild hypointense HCC lesions (arrow) (Figure 2A). On corresponding coronal HASTE (T2-weighted) images, liver tumors are difficult to distinguish from adjacent liver parenchyma because of signal isointensity (Figure 2B). Fat-suppressed post-Gd SGE imaging showed an almost homogenous signal (arrows) of moderately enhancing liver tumors (Figure 2C). Three weeks after onset of sorafenib therapy, nonenhanced T1WI imaging revealed multiple focal strongly hyperintense lesions (arrows) presumed to represent hemorrhagic necrosis in the known tumors (Figure 2D). Coronal HASTE images performed at the same time demonstrated also hyperintense signals (arrows) of tumors with a good delineation to adjacent liver parenchyma (Figure 2E). Fat-suppressed post-Gd SGE imaging showed central >75% necrosis in most of the HCC lesions following sorafenib therapy (Figure 2F).

**Figure 3:**
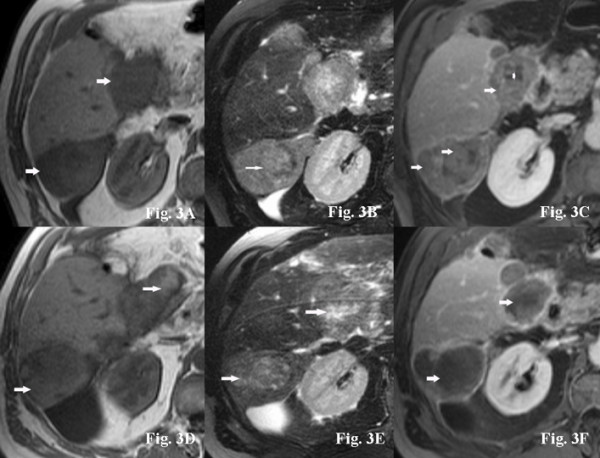
**A-F. 61-year-old male patient with metastatic HCC (patient #17)**. Axial nonenhanced T1WI imaging of the liver showed hypointense signal of all HCC lesions (arrows) (Figure 3A). On corresponding T2WI images, HCC lesions demonstrated all a moderate hyperintense signal (arrows) compared to adjacent liver parenchyma (Figure 3B). Fat-suppressed post-Gd SGE imaging revealed a diffuse enhancement throughout all HCC lesions (arrows) with small central areas of necrosis (arrowhead) (Figure 3C). Five weeks after onset of sorafenib therapy, the signal of HCC lesions on T1WI- imaging increased (arrows) becoming iso- to hyperintense to adjacent liver parenchyma (Figure 3D). At this time, there was at best mild increase in tumor signal on fat-suppressed T2WI imaging (arrows) (Figure 3E). However, fat-suppressed post-Gd T1WI demonstrated >75% reduction of enhancing tumor areas due to necrosis (arrows) (Figure 3F).

Typical examples of signal intensity changes of MRI baseline imaging and follow-up investigations are presented in Figure 1, 2 and 3 for three representative patients (patients #7, #8, and #17). In Figure 1, typical changes from a patient presenting with a T1WI hypointense tumor signal at baseline and consecutive changes under therapy are shown. Figure 2 demonstrates synchronous hemorrhagic necrosis in all hepatic lesions of a patient with multicentric HCC 3 weeks after onset of sorafenib therapy. Figure 3 demonstrates increased signals on T1WI and T2WI occurring synchronously in all HCC manifestations after onset of sorafenib therapy. Of note, one patient (#21) presented a slow but continuous growth of the hepatic lesions despite typical signal changes on T1WI and T2WI. This patient had repeated therapy interruptions and a dose reduction due to a hand-foot syndrome, but a repeated increase in T1WI was observed following the re-onset of sorafenib. Two patients in this series, who showed a continuous tumor growth under treatment (#11, #18), showed no sorafenib related signal abnormalities on MRI at all and no *de novo *occurring or progressive necrosis.

### Potential Influence of Sorafenib Dose on Tumor Signal

Thus, early changes occurred in the 8 patients that were able to take sorafenib 800 mg daily within a mean auf 4.1 weeks (range, 3–5). Taking all 18 patients together who received either sorafenib 800 mg or 400 mg daily, the latter being the first dose reduction level, early changes could be detected within a mean of 4.2 weeks (range, 2–9), demonstrating that both dose levels had a similar reaction pattern. However, in a further patient who had to reduce sorafenib to 200 mg due to adverse reactions early in the therapy course, MRI signal alterations and new tumor necrosis could be detected at week 12 under therapy. Of note, the 2 patients with a longer duration until a signal response could be detected (#3, #16) had to temporarily discontinue therapy within the first two weeks due to a grade 3 skin reaction or a transient increase in bilirubin levels, respectively.

In conclusion, we show for the first time that rapid changes in MR signal intensities occur soon after onset of sorafenib therapy. A statistical analysis addressing the presence or absence of an increased T1WI signal, tumor necrosis, and increase in the necrotic volume in relation to the entire tumor volume [NV/TV] showed a significant relationship between T1WI and tumor necrosis (p = 0.017) and between tumor necrosis and NV/TV (p = 0.002). The direct association between T1WI and NV/TV did not reach statistical significance (p = 0.129) in this small sample size

## Discussion

Targeted therapy refers to a new generation of anticancer drugs, designed to interfere with distinct molecular entities, involved in tumor growth and/or progression. The multikinase inhibitor sorafenib demonstrated profound anti-tumor activity in different clinical settings, including HCC [5,6,17], renal cell carcinoma (RCC) [9,18], as well as other advanced, so far therapy-refractory tumor entities [19,20]. In the special situation of HCC, imaging studies have to take into account that tumor areas develop within a heavily diseased liver, in which several clonal tumor areas with different patterns of differentiation might exist simultaneously [2,3].

In our patient population a substantial impact of sorafenib on the MRI pattern of HCC tissues was observed, which might be regarded at least as some kind of a therapy response; however, the majority of these changes did not lead to a classification of a partial response according to classical RECIST criteria. Nevertheless, 16 out of 21 patients presented with either new or progressive necrosis in repeated MRI scans, which suggests that quantifying necrotic areas in relation to the entire tumor volume might be useful as a reliable predictive marker of therapy response. Similar results have been described for 11 HCC patients under sorafenib treatment using a semiautomated computerized technique to analyse tumor necrosis in contrast enhanced CT scans [5]. However, the influence of tumor necrosis on the clinical outcome, including overall survival data, has not been prospectively investigated yet.

Reaction patterns to targeted agents like sorafenib seem to foremost include (i) disease stabilization (rather than a direct cytotoxic effect accompanied by tumor shrinkage) or (ii) an induction of intralesional necrosis that does not automatically lead to a marked decrease in tumor size. In this context, a surprising finding of our study was that more than one third of the tumors that displayed new necrotic areas under treatment showed a temporary expansion, which seemed to be primarily due to an increased volume induced by tumor necrosis and not by an accumulation of vital tumor cells. Interestingly, comparable data have been reported in patients with gastrointestinal stromal tumors (GIST), a different tissue context receiving therapy with the tyrosine kinase inhibitor imatinib mesylate (STI571; Gleevec™) [21]. This information must be taken into account when judging tumor responses under treatment with both, single kinase (e.g. imatinib mesylate) as well as multikinase inhibitors (e.g. sorafenib).

Especially in the context of HCC, MRI provides a highly sensitive method for detecting soft tissue signal changes, to assess the extent of therapy-related tumor necrosis, and to monitor both, distinct hepatoma nodules as well as the surrounding liver parenchyma in exquisite detail [22]. It is obvious that signal abnormalities related to sorafenib that occur at a distinct time point are only temporary in nature because of a continuous alteration of therapy induced signal abnormalities, e.g. by physiologic processes, leading to hemoglobin degradation or change in the tissue protein content. Both protein-rich secretion due to rapid tumor necrosis or hemorrhage could explain signal abnormalities on MRI. However, taken all signal abnormalities occurring on T1WI and T2WI and their temporary evolution into account, hemorrhage seems to be much more plausible. Theoretically, looking at an early time point soon after the initiation of a targeted therapy, the appearance of intracellular deoxyhemoglobin is expected to induce low signal intensities on both T1WI and T2WI, similar to well-known signal abnormalities, occurring early in acute hemorrhage due to other causes (days 1 – 3 after occurrence). However, detection of such changes is expected to be less sensitive in tumors that present initially with either isointense signals or signal intensities that are only slightly different from that of adjacent liver parenchyma and would require a very early monitoring, for instance by MR-imaging, which was not the focus of our study. In the subsequent early subacute phase (> 3 days after onset of hemorrhage), the transformation of intracellular deoxyhemoglobin to methemoglobin is known to induce a change of the MR-signal to high intensities on T1WI and low intensities on T2WI images, whereas in the late subacute phase (> 7 days post hemorrhage), the occurrence of extracellular methemoglobin results in high signals in both T1WI and T2WI, which contrasts much better with the native tumor and liver parenchymal signal. This time span (first weeks after initiation of therapy) seems to be most adequate for accurate demonstration of hemorrhage in tumors as caused by sorafenib and corresponds to our imaging schedule. Other theoretical causes for an increase of signal intensity on nonenhanced T1WI include a protein-rich necrosis, similar to a coagulation necrosis that occurs after radiofrequency ablation. However, in that setting, the T2WI signal of those lesions is expected to be low and persists for a much longer time period than hemorrhagic alterations observed in our sorafenib treated patients. Nevertheless, the exact time required for the reconversion of MR signals to baseline values could not be reliably determined, as the influence of variable sorafenib dosages remains unknown.

Alternative explanations for signal abnormalities occurring during sorafenib treatment in our cohort include tumor hemorrhage or necrosis induced by tumor progression. However, the latter could indirectly be excluded by further follow-up MR imaging studies, which demonstrated either tumor size stabilization or more often a continuously rising ratio of the volume of necrotic tumor areas to viable tumor tissue.

There are some limitations of our work, first of all caused by the small number of patients recruited and evaluated. Follow-up studies with larger numbers of patients are needed in order to validate our results and establish the accuracy of such a response assessment. Secondly, for scientific reasons it would be desirable to compare radiological signal changes under therapy with histological alterations. However, due to ethical considerations, such a parallel evaluation is not feasible in humans. Nevertheless, long-term monitoring as in our study is regarded as an acceptable alternative.

## Conclusion

In summary, early changes within T1WI, T2WI, Gd-enhancement and necrotic tumor areas seem to define rapid therapy responses in HCC patients receiving sorafenib. Due to the observation that an induction of intralesional necrosis does not automatically correlate with a decrease in tumor size, the application of classical RECIST criteria may not be suitable to identify patients who benefit from the sorafenib therapy. Therefore, the prospective value of early MRI changes within one or several of the investigated parameters and a predefined time frame, with respect e.g. to a survival benefit should be evaluated prospectively in subsequent larger clinical trials. Prognostic parameters being available within weeks of therapy initiation could be beneficial in two ways: (i) MR-imaging could lead to an early change of the therapeutic strategy and thus influence the outcome in these HCC patients; (ii) severe side effects as well as a substantial amount of costs potentially could be avoided in well-defined non-responders.

## Competing interests

The authors declare that they have no competing interests.

## Authors' contributions

MB, CPB, UML, and MG enrolled and took care of the investigated patients in this observational study; MH, CS, and CCC analysed the radiological scans; UK, MB, and UML gathered and interpreted the clinical data; MH, UML, and MB were involved in drafting the manuscript. All authors have read and approved the final manuscript.

## Pre-publication history

The pre-publication history for this paper can be accessed here:

http://www.biomedcentral.com/1471-2407/9/208/prepub
